# Diversity in breeding sites and distribution of *Anopheles* mosquitoes in selected urban areas of southern Ghana

**DOI:** 10.1186/s13071-016-1941-3

**Published:** 2017-01-13

**Authors:** Precious A. Dzorgbe Mattah, Godfred Futagbi, Leonard K. Amekudzi, Memuna M. Mattah, Dziedzorm K. de Souza, Worlasi D. Kartey-Attipoe, Langbong Bimi, Michael D. Wilson

**Affiliations:** 1 Institute of Environment and Sanitation Studies (IESS), University of Ghana, Legon, Ghana; 2 Directorate of Academic Planning and Quality Assurance (DAPQA), University of Cape Coast, Cape Coast, Ghana; 3 Department of Animal Biology and Conservation Science, University of Ghana, Legon, Ghana; 4 Department of Physics, Kwame Nkrumah University of Science and Technology, Kumasi, Ghana; 5 Department of Environment and Development Studies, Central University, Accra, Ghana; 6 Parasitology Department, Noguchi Memorial Institute of Medical Research, University of Ghana, Legon, Ghana

**Keywords:** *Anopheles*, Urban, Distribution, Permanent habitat, Temporary habitat, Breeding site

## Abstract

**Background:**

*Anopheles* vectors of malaria are supposedly less common in urban areas as a result of pollution, but there is increasing evidence of their adaptation to organically polluted water bodies. This study characterized the breeding habitats of *Anopheles* mosquitoes in the two major urban areas in southern Ghana; Accra (AMA) and Sekondi-Takoradi (STMA) Metropolitan Areas, during dry and wet seasons.

**Methods:**

*Anopheles* mosquito larvae were sampled using standard dipping methods to determine larval densities. The origin, nature and stability of 21 randomly selected sites were observed and recorded. Mosquito larvae were reared to adults and *Anopheles* species identified by both morphological and molecular means.

**Results:**

Sixty-six percent of *Anopheles* habitats were permanent and 34% temporal, and 74.5% man-made while 25.5% were natural. Puddles and urban farm sites accounted for over 51% of all *Anopheles* mosquitoes sampled. The mean larval densities among the habitat types was highest of 13.7/dip for puddles and lowest of 2.3/dip for stream/river, and the variation between densities were significant (*P* = 0.002). The mean larval densities were significantly higher in the wet season than in the dry season for the two study areas combined (*P* = 0.0191) and AMA (*P* = 0.0228). Over 99% of the 5,802 morphologically identified *Anopheles* species were *An. gambiae* (*s.l.*) of which more than 99% of the studied 898 were *An. coluzzii* (62%) and *An. gambiae* (*s.s*.) (34%). Urban farms, puddles, swamps and ditches/ dugouts accounted for approximately 70% of all *An. coluzzii* identified. Conversely, drains, construction sites, streams/rivers and “others” contributed 80% of all *An. gambiae* (*s.s*.) sampled. The wet season had significantly higher proportion of *Anopheles* larvae compared to the dry season (*Z* = 8.3683, *P* < 0.0001). Also, the proportion of *Anopheles* mosquitoes produced by permanent breeding sites was 61.3% and that of temporary sites was 38.7%.

**Conclusion:**

Taken together, the data suggest that man-made and/ or permanent habitats were the main contributors to *Anopheles* larval populations in the cities and that regulation of the anthropogenic processes that lead to development of breeding places and proper environmental management can drastically reduce mosquito breeding sites in urban areas of Ghana.

**Electronic supplementary material:**

The online version of this article (doi:10.1186/s13071-016-1941-3) contains supplementary material, which is available to authorized users.

## Background

There is a growing interest in urban malaria in sub-Saharan Africa (SSA). This is because factors which support the prevalence of malaria in urban areas are mainly anthropogenic and are those that promote the continuous presence, breeding and propagation of *Anopheles* mosquitoes and malaria parasites in the cities [1]. Although increasing surface water pollution caused by rapid urbanization was believed to have hampered the development of *Anopheles* larvae and eliminate certain species like *Anopheles funestus*, others such as species of the *Anopheles gambiae* complex have adapted well and continue to breed even in organically polluted water bodies [2, 3]. This is contrary to the conventional view that *Anopheles* mosquitoes breed only in clean or clear water habitats [4, 5].

*Anopheles* mosquitoes exploit varying habitats for breeding. They breed in and around vicinities of deteriorating infrastructure such as broken water pipes, open tins/cans, poorly maintained drains, culverts, market gardens/urban agricultural sites, pools at construction sites, lorry tyre tracks on unpaved roads, low lying areas that are liable to flooding, hydrants, catch pits among others. These are all found close to or in-between houses in the city [6–8]. Most *Anopheles* mosquito breeding habitats in urban areas are man-made even though natural water habitats are found scattered around the urban milieu [9]. Perceivably, all available landscapes within the city, whether natural or man-made, that collect any form of stagnant water are potential breeding places for mosquitoes and may possibly be habitats for *Anopheles* mosquitoes. Such habitats are most often maintained in the city by human activities which have underneath it, poor hygiene practices supported by lack of sanitation facilities and poor maintenance culture [10]. Several studies on *Anopheles* breeding habitats in urban areas of Africa, characterized the breeding places [3, 7, 9]. It has been observed that urbanization could influence the epidemiological characteristics of diseases through the provision of good breeding environment for vectors of infectious diseases [11].

Already, among all the tropical diseases, malaria is considered the most common and devastating especially in SSA. In Ghana, malaria transmission is an all-year round phenomenon with peaks in the rainy seasons, and accounts for between 30–40% of outpatient visits to health facilities each year [12–14]. In Accra, malaria constitutes 40% of outpatient visits in most health facilities [15, 16], a higher figure compared to the national average. This study aims at determining habitat preference of *Anopheles* mosquitoes in Accra and Sekondi-Takoradi municipalities of Ghana. We characterized the breeding places of *Anopheles* mosquitoes by origin, including man-made or naturally occurring and whether temporary or permanent, in 21 randomly selected points. Natural habitats were considered as naturally occurring, and man-made habitats were those created by anthropogenic activities. Water bodies in which larvae were found at least once and which dried up at least once during the sampling period were classified as temporary. We also defined a permanent habitat as the one in which *Anopheles* larva were found at least once and contain water throughout the sampling period. Habitat diversity and the types of *Anopheles* mosquito breeding in these habitats were also studied. In light of the recent reclassification of the M and S molecular forms of *An. gambiae* (*s.s.*), as *An. coluzzii* and *An. gambiae* (*s.s.*), respectively, it was found necessary to determine their habitat preferences in urban settings.

## Methods

### Study areas

**Fig. 1:**
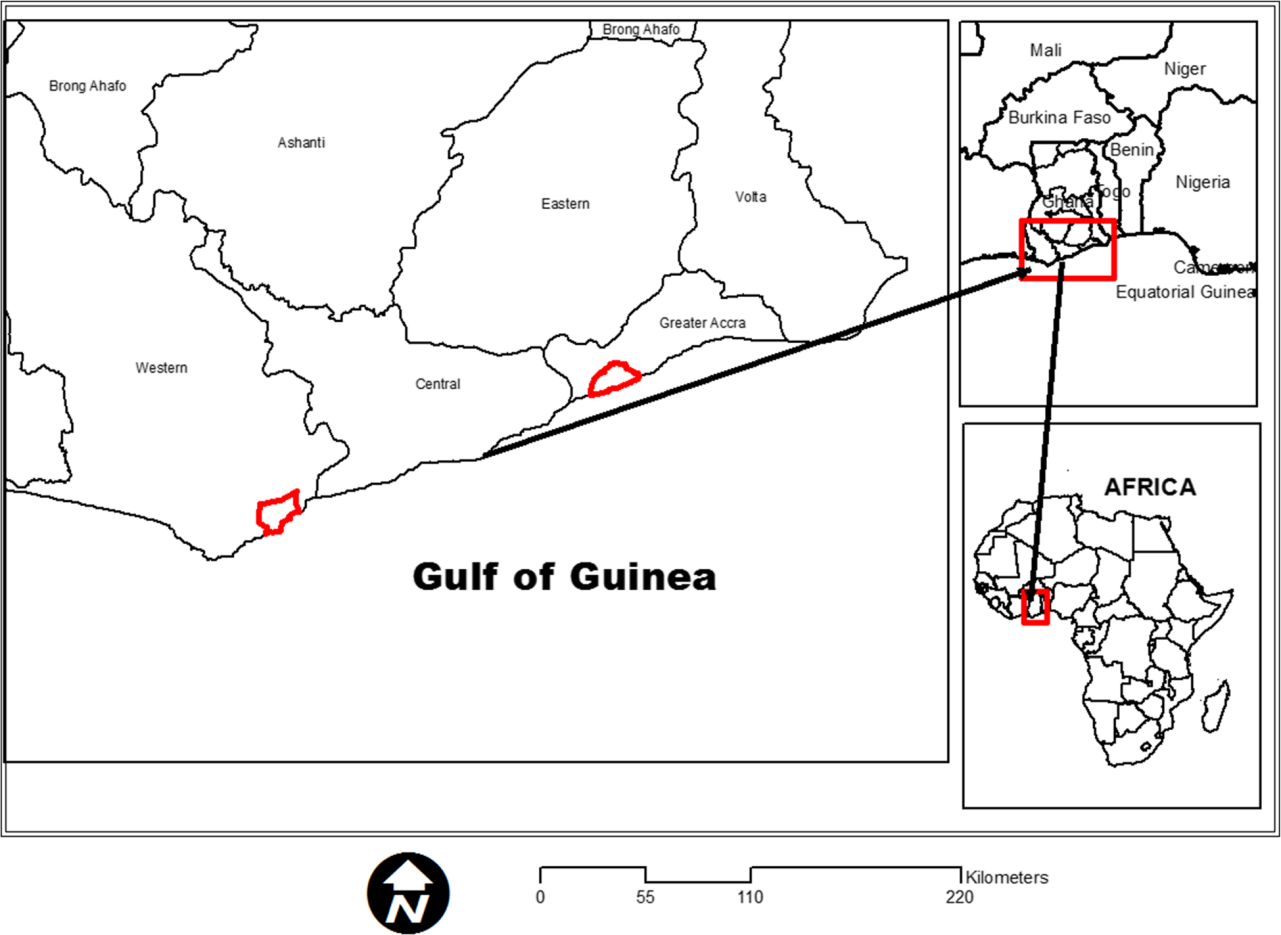
Location of the study areas in southern Ghana

The study was conducted in the two major and the most populous urban areas of coastal Ghana; Accra Metropolitan Area (AMA) and the Sekondi-Takoradi Metropolitan Area (STMA) (Fig. 1). AMA is the national capital and STMA is a regional capital, a harbour city and a hub of activities related to the offshore oil production in the Western Region of Ghana. In 2010, AMA has an approximate population of 1.7 million while that of STMA was 445,205 [17]. The two cities also have and continue to attract migrants from both within and outside the country. Though these two metropolitan areas are in the same ecological zone-coastal savanna zone [17], STMA which is at the western edge of this ecological zone exhibits more characteristics of the rainforest belt and also experience more rainfall than AMA. The climate of southern Ghana is tropical, characterized by two distinctive rainfall seasons- a major one between April and June and a minor one which occurs between September and October. Relative humidity is generally high over 65%.

### Selection of sampling sites

**Fig. 2:**
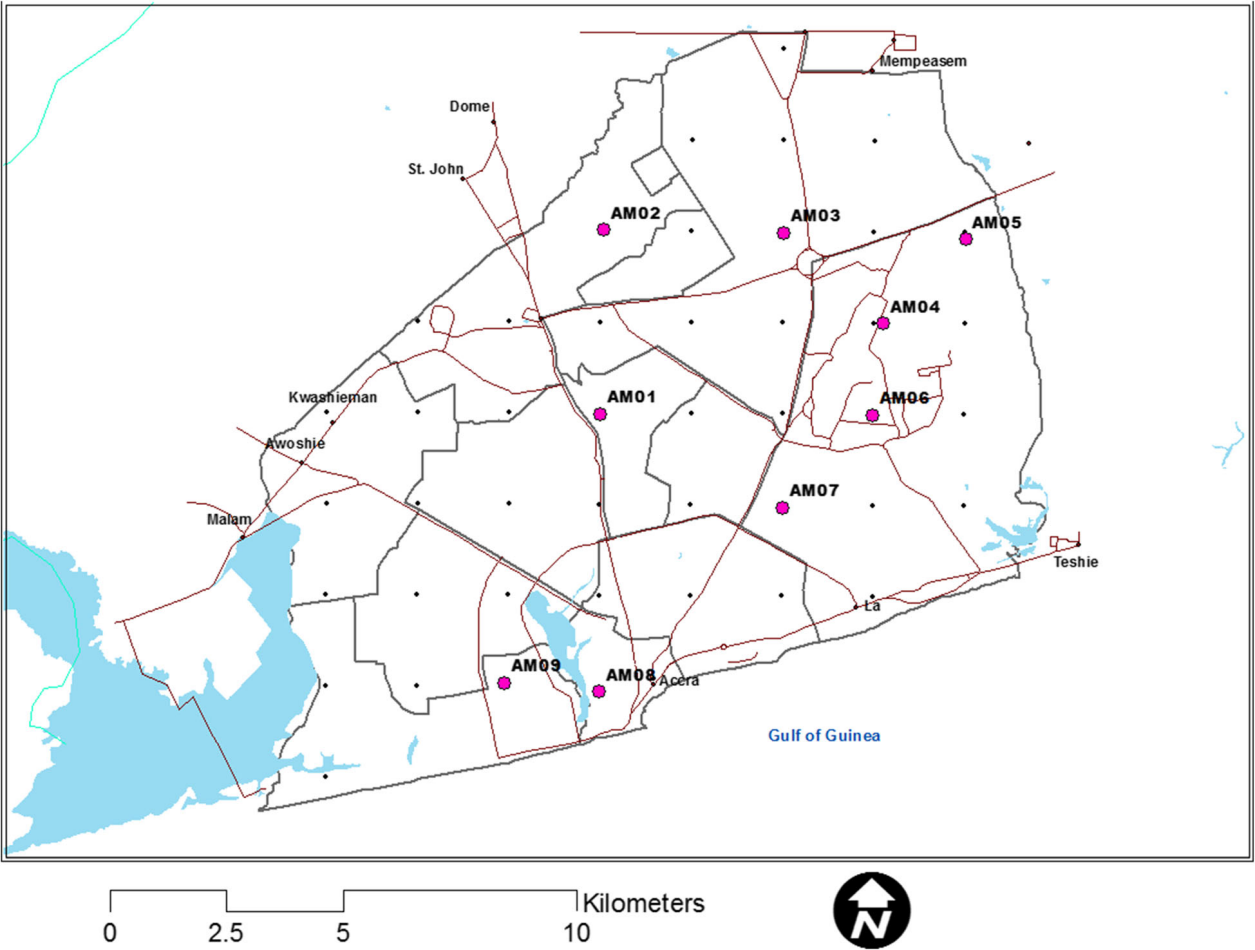
Sampling points in Accra Metropolitan Area

**Fig. 3:**
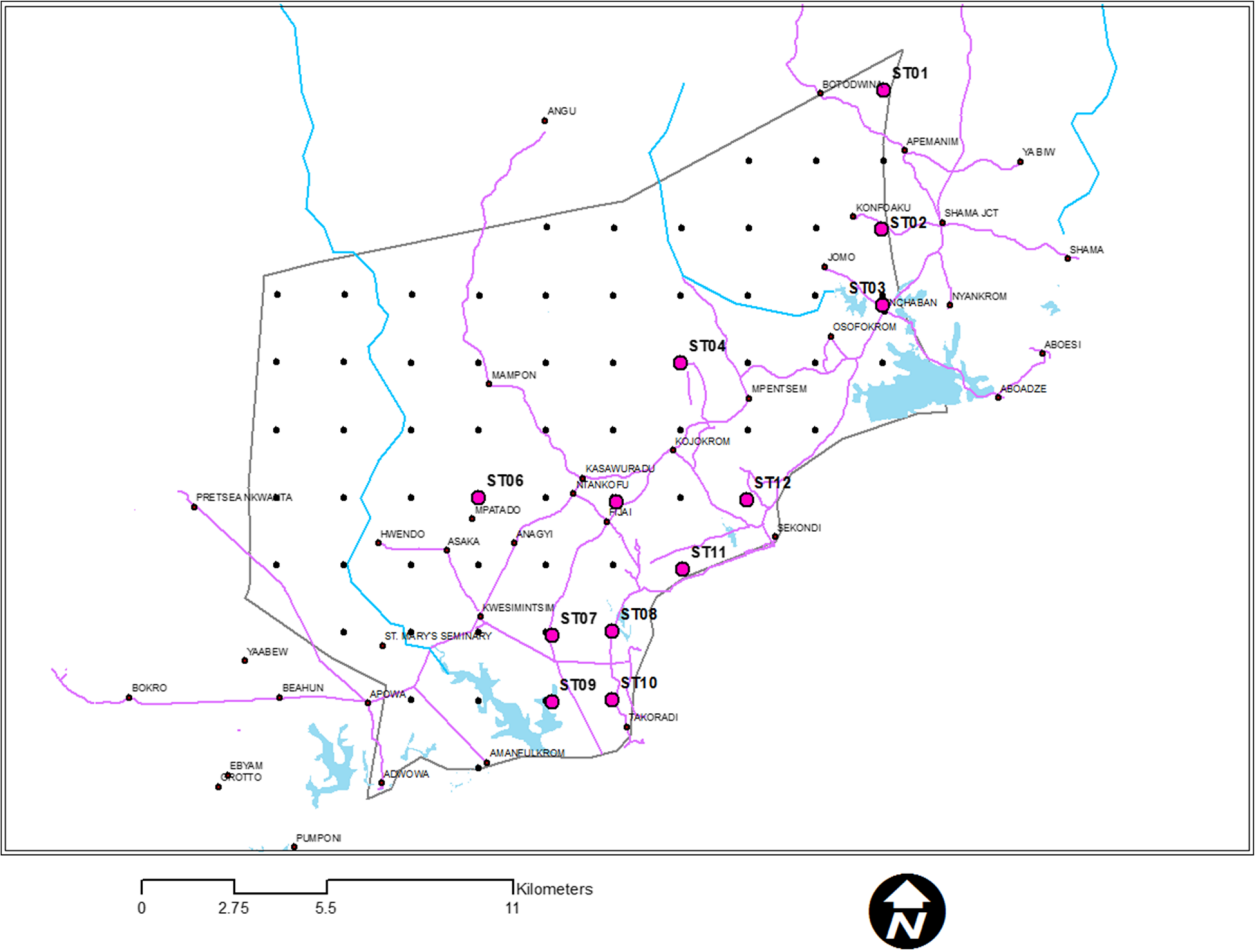
Sampling points in Sekondi-Takoradi Metropolitan Area

Using the R software 3.0.1 (R Core Team, 2012) and ArcGIS (ESRI, Redlands, California, USA) maps of the study areas were divided by an overlaid grid of 2 km (a homogeneous distance that could cater for land use and land cover changes in urban areas). The grids were converted to point features through the determination of the centroid (central point) of each of the grids. The points were provided with unique numbers and constituted a sampling frame from which 20% was sampled using the random sample of cases in SPSS version 16 (SPSS Inc Chicago, USA). The final sampled points were geo-referenced and their locations (longitudes and latitudes) noted and stored in a GPS for further identification on the field. Where a selected point was not accessible, nearest accessible point was used to replace it. In all, a total of 9 points were sampled out of 44 that constituted the sampling frame for AMA and 12 points sampled out of 62 for STMA (Figs. 2 and 3).

### Characterization of *Anopheles* breeding sites

Habitats of the species were described with respect to whether they were temporary or permanent and man-made or natural.

### *Anopheles* larval sampling

Before the fieldwork, a reconnaissance survey was conducted during which the sampling points were located on the field using a Garmin GPS navigator (Garmin Inc., Kansas, USA). Identification of *Anopheles* mosquito breeding sites was achieved through once-a-month larval sampling for 11 consecutive months at the sampling points. Surroundings (within 100 m radius) of the sampling points were thoroughly searched for possible breeding sites of *Anopheles* mosquitoes. All identified water bodies likely to harbour mosquito larvae were visually searched thoroughly for the presence of *Anopheles* mosquito larvae. The presence of *Anopheles* larvae was determined after 15 dips. Sampling of *Anopheles* larvae was done using the standard dipping method [18]. A standard dipper 350 ml (Bio Quip Products Inc., Gardena, California, USA) was used. The area of each water body was calculated by walking around the water body (if possible) using a handheld GPS map 62 navigator (Garmin). With large water bodies usually (> 10 m^2^), 10 dips per habitat was taken at eight different points. Six dips were taken at 4 different points in small water bodies (< 10 m^2^). For this study, a dip represents a volume of 350 ml*.* Larvae sampled were collected in well-labelled plastic containers (whose covers were perforated to allow for ventilation) and transported to an insectary for rearing to maturity and identification.

### Identification of *Anopheles* mosquitoes

Larvae brought to the insectary were reared to adults using the methods provided by the Malaria Research and Reference Reagents Resource (MR4) [19]. All adult *Anopheles* mosquitoes were identified morphologically using the keys of Gillies & De Meillon [20] and Gillies & Coetze [21].

To identify the sibling species of the *Anopheles gambiae* complex, genomic DNA of the mosquitoes was extracted using the boiling method [22]. The PCR method proposed by Scott et al. [23] was used to allow for simultaneous identification of the members of *Anopheles gambiae* complex. With this method, species-specific oligonucleotide primers were used to identify *An. gambiae* (*s.s.*)*, An. melas* and *An. arabiensis*. The primer sequence details and the expected sizes of the PCR products are: Universal (UN) 5′-GTG TGC CCC TTC CTC GAT GT-3′ at 56 °C and 468 bp; *An. gambiae* (GA) 5′-CTG GTT TGG TCG GCA CGT TT-3′ at 62 °C and 390 bp; *An. melas* (ME) 5′-TGA CCA ACC CAC TCC CTT GA-3′ at 90 °C and 464 bp; *An. arabiensis* (AR) 5′-AAG TGT CCT TCT CCA TCC TA-3′ at 78 °C and 315 bp; *An. quadrannilatus* 5′-CAG ACC AAG ATG GTT AGT AT-3′ at 54 °C and 153 bp.

The total volume of PCR reaction mix was 25 μl, containing 1× PCR buffer supplied by the manufacturer (Sigma-Aldrich, St. Louis, USA), 20 μM deoxyribonucleotide triphosphates (dNTPs), 10 μM of each of the four oligonucleotide primers (UN, ME, GA, AR), 0.10 Units of *Taq* polymerase (Sigma-Aldrich, St. Louis, USA) and 2.5 μl of the extracted DNA. Sterile double distilled water was used to make up the volume of 25 μl. The reaction mix was centrifuged briefly and overlaid with mineral oil to avoid evaporation and refluxing during thermo-cycling. A PTC 100 thermal cycler (MJ Research Inc., Waltham, USA) was used to amplify the DNA. The cycling process involved an initial denaturation at 95 °C for 10 min, followed by 30 cycles of denaturation at 94 °C for 30 s, annealing at 50 °C for 30 s and extension at 72 °C for 30 s. A final extension at 72 °C for 7 min was performed. For each reaction, negative and positive controls containing no DNA template and a known DNA template were respectively added.

The amplified products were analysed by agarose gel electrophoresis, using 7 μl of each PCR product and electrophoresed in 2% agarose gel stained with 0.5 μg/ml of ethidium bromide (EtBr). The electrophoresis was run in 1X Tris acetate- EDTA (TAE) buffer at 100 V for one hour and the gel visualized over a UV transilluminator (Labnet International Inc, Edison, USA). The sizes of the PCR products were estimated by comparison with a 100 bp DNA molecular weight ladder (Biolabs Inc., New England, USA). The separation of *Anopheles gambiae* (*s.s.*) from *Anopheles coluzzii* was done using polymerase chain reaction-restriction fragment length polymorphism (PCR-RFLP) method of Fanello et al. [24]. Ten (10) μl of PCR product was combined with 1U of *Hha*1 enzyme (Promega Corporation, Madison, USA) in 10× enzyme buffer (Promega Corporation, Madison, USA).

### Statistical analysis

Data were captured, managed and analysed using the Statistical Package for Social Sciences (SPSS version 16, Inc., Chicago, IL, USA) and Graph Prism Statistical software (Prism, GraphPad Software, San Diego, CA, USA). One-way Analysis of Variance (ANOVA) was used to compare variations in larval densities and counts among habitat types. Pairwise comparisons of larval densities or counts were carried out using Tukey’s and Dunnett’s tests and *Z*-test for proportions.

## Results

### Habitat diversity of sampling areas

Habitat types were characterised in each sampling area. Only one of the 21 sampling points did not have any water body within its 100 m radius during the sampling period. Water bodies found were grouped into eight different habitat types including puddles, swamps, drains (paved and unpaved), ditches/dugouts, construction sites, urban farm sites, streams/river edges and “others” (lorry tyres and containers). Puddles and paved drains were most common and were both found in eight of the sampling points. Other habitats such as abandoned lorry tyres and containers filled with rainwater were found in five of the sampling points (Table 1).

**Table 1:** Frequency of habitat types and their contribution to total *Anopheles* mosquitoes sampled in Accra and Sekondi-Takoradi Metropolitan Areas

	Frequency at sampling points *n* (%)	Contribution to total *Anopheles* mosquitoes sampled (%)		
		AMA	STMA	ALL
Puddles	8 (19.0)	36.9	16.4	28.6
Swamps	4 (9.5)	1.3	30.6	13.2
Paved drains	8 (19.0)	12.4	11.5	12.1
Unpaved drains	3 (7.1)	6.2	–	3.7
Ditches/dugouts	4 (9.5)	0.6	9.3	4.2
Construction sites	3 (7.1)	10.6	6.6	9.0
Urban farms	3 (7.1)	23.9	22.8	23.2
Streams/rivers	3 (7.1)	5.0	2.8	4.2
Others	5 (11.9)	3.1	–	1.9

*Abbreviations*: *AMA* Accra Metropolitan Area, *STMA* Sekondi-Takoradi Metropolitan Area

In 80.57% of 242 visits made throughout the study period, water was found in various habitat types within the 100 m radius of sampling points. Most, 161 (82.8%) of 195 habitats were breeding places for different types of mosquitoes. However, *Anopheles* mosquito larvae were found in 79 (49%) of the 161 breeding places. In AMA, 46 (66%) of 70 habitats contained *Anopheles* mosquitoes while 48 (38%) of 125 habitats in STMA had *Anopheles* mosquitoes. Thus the frequency of seeing a habitat harbouring *Anopheles* larvae in AMA was higher (1.7 times) than in STMA.

Puddles and urban farm sites in the two cities accounted for more than 51% of all *Anopheles* mosquitoes sampled (28.6 and 23.2%, respectively; Table 1). Habitat types such as old lorry tyres and open containers which were grouped under “others” contributed only 1.9% of all *Anopheles* mosquitoes sampled. In AMA, the same two habitats, puddles and urban farm sites, contributed about 61% (36.9 and 23.9%, respectively). The situation was however different in STMA where swamps and urban farm sites contributed approximately 53% (30.6 and 22.8%, respectively) to the total *Anopheles* mosquitoes sampled during the period. Ponds/lagoons had no *Anopheles* mosquitoes and unpaved drains and “others” were not found in STMA.

### Distribution of *Anopheles* mosquitoes in the study areas

Five thousand eight hundred and two larvae were reared to adults. Exactly 99.9% of these were identified morphologically were *An. gambiae* (*s.l.*). All *Anopheles* mosquitoes sampled in AMA were *An. gambiae* (*s.l.*) while in STMA, *Anopheles* species such as *An. coustani* (0.06%) and *An. rufipes* (0.05%) were also found (Table 2).

**Table 2:** Proportions of members of *Anopheles gambiae* complex identified in Accra and Sekondi-Takoradi Metropolitan Areas

		AMA	STMA	ALL
		*n* (%)	*n* (%)	*n* (%)
Number of each *Anopheles* spp. (%)	*An. rufipes*	0 (0)	3 (0.14)	3 (0.05)
	*An. coustani*	0 (0)	4 (0.18)	4 (0.06)
	*An. gambiae* (*s.l.*)	3,564 (100.00)	2,224 (99.68)	5,795 (99.87)
Number of each *Anopheles* (*s.l.*) members (%)	*An. melas*	0 (0)	2 (0.50)	2 (0.20)
	*An. coluzzii*	225 (43.80)	328 (85.86)	553 (61.72)
	*An. gambiae* (*s.s.*)	234 (45.50)	24 (6.28)	258 (28.79)
	Unidentified	55 (10.70)	30 (7.85)	85 (9.48)

*Abbreviations*: *AMA* Accra Metropolitan Area, *STMA* Sekondi-Takoradi Metropolitan Area

Using molecular techniques, 898 of the *An. gambiae* (*s.l.*) from both cities were further identified as *An. coluzzii, An. gambiae* (*s.s.*) and *An. melas. Anopheles coluzzii* and *An. gambiae* (*s.s.*) accounted for 99.8% of the *An. gambiae* (*s.l.*). Only two (0.2%) of the *An. gambiae* (*s.l.*) were identified as *An. melas* and these species were found only in STMA. Restrictions digest on 896 *An. gambiae* (*s.l.*) from all the study areas revealed that *An. coluzzii* was the majority (62%) and about 9% could not be identified after two repetitions of the restrictions digest (﻿Additional file 1). In AMA, there was no significant difference between proportions of *An. coluzzii* and *An. gambiae* (*s.s.*) (*Z* = 0.7235, *P* = 4694). In STMA, however, the proportion of *An. coluzzii* was significantly and strikingly higher than that of *An. gambiae* (*s.s.*) (*Z* = 41.5392, *P* < 0.0001).

### Temporal distribution of *Anopheles* species

**Fig. 4:**
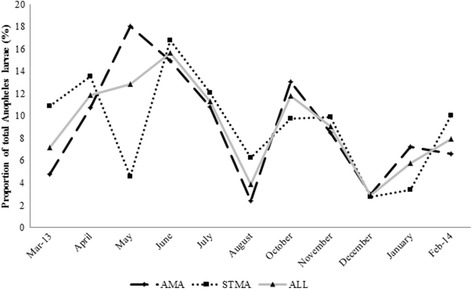
Monthly distribution in the proportion of *Anopheles* larvae in Accra (AMA) and Sekondi-Takoradi Metropolitan Areas (STMA). Line ALL indicates monthly distribution of total *Anopheles* larvae sampled from both cities

Overall, monthly counts of *Anopheles* mosquitoes sampled followed a bi-modal trend with peaks in June and October. The two peak months accounted for 15 and 11.8%, respectively, of all *Anopheles* mosquitoes sampled (Fig. 4). Coincidentally, June and October were periods when high numbers, 14 (67%) and 11 (52%) respectively, of the 21 sampling points had breeding places with larvae. Two dips were observed in the proportion of *Anopheles* mosquitoes sampled in August (3.9%) and December (2.9%) and these also occurred when fewer (28 and 19%, respectively) sampling points had breeding places. A similar trend was observed in AMA with the peak proportions of *Anopheles* mosquitoes occurring in May (18%) and October (13%). Three dips were also observed in AMA in March, August and December with that of August being the lowest (2.4%). Monthly distributions in *Anopheles* larvae sampled in STMA however were characterized with fluctuations with three peaks in April, June and November as well as three dips in May, August and December.

**Fig. 5:**
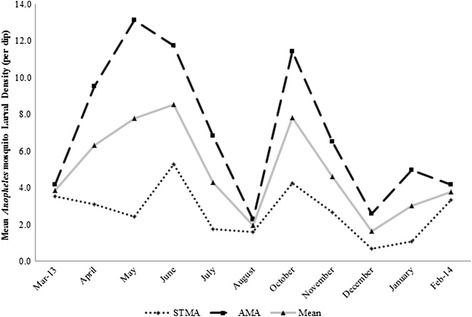
Monthly distribution of *Anopheles* larval density in Accra (AMA) and Sekondi-Takoradi Metropolitan Areas (STMA). Line ALL indicates monthly distribution of pooled *Anopheles* larval density for both cities

Mean monthly larval density was also characterized with double maxima in the months of June (8.5/dip) and October (7.8/dip) for the entire study areas. Mean monthly larval densities were generally higher for AMA than STMA except for the month of August in which both AMA and STMA recorded an approximate mean of 2.0/dip (Fig. 5). The highest larval density (13.1/dip) for AMA was recorded in May, followed by October (11.4/dip).

**Fig. 6:**
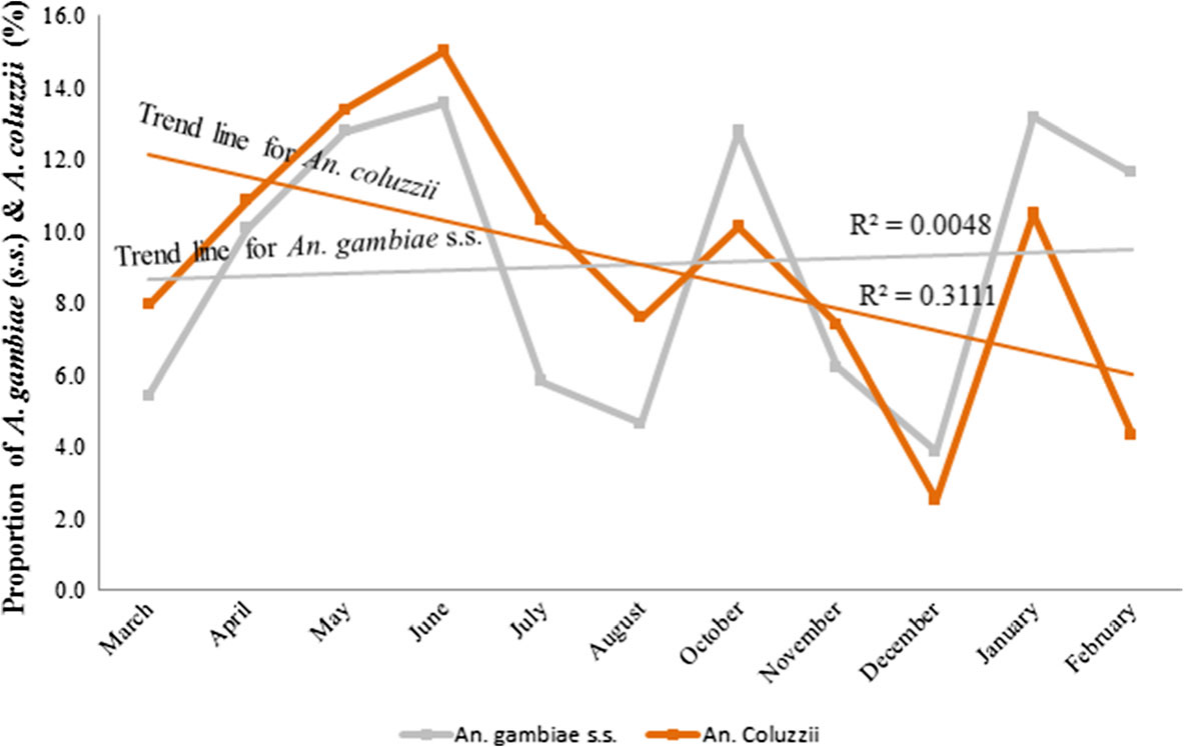
Monthly distribution of *An. coluzzii* and *An. gambiae* (*s.s.*)

The overall monthly distribution of *An. coluzzii* and *An. gambiae* (*s.s.*)*,* in both cities, had *An. coluzzii* predominating in seven months, including March, April, May, June, July, August and November (Fig. 6). Proportions of *An. gambiae* (*s.s.*) were high in the months of October and December, 2013, and January and February, 2014. Even though the population of both species fluctuates throughout the year, *An. coluzzii* generally declined from March 2013 to February 2014 while *An. gambiae* (*s.s.*) increased slightly over the same period.

**Fig. 7:**
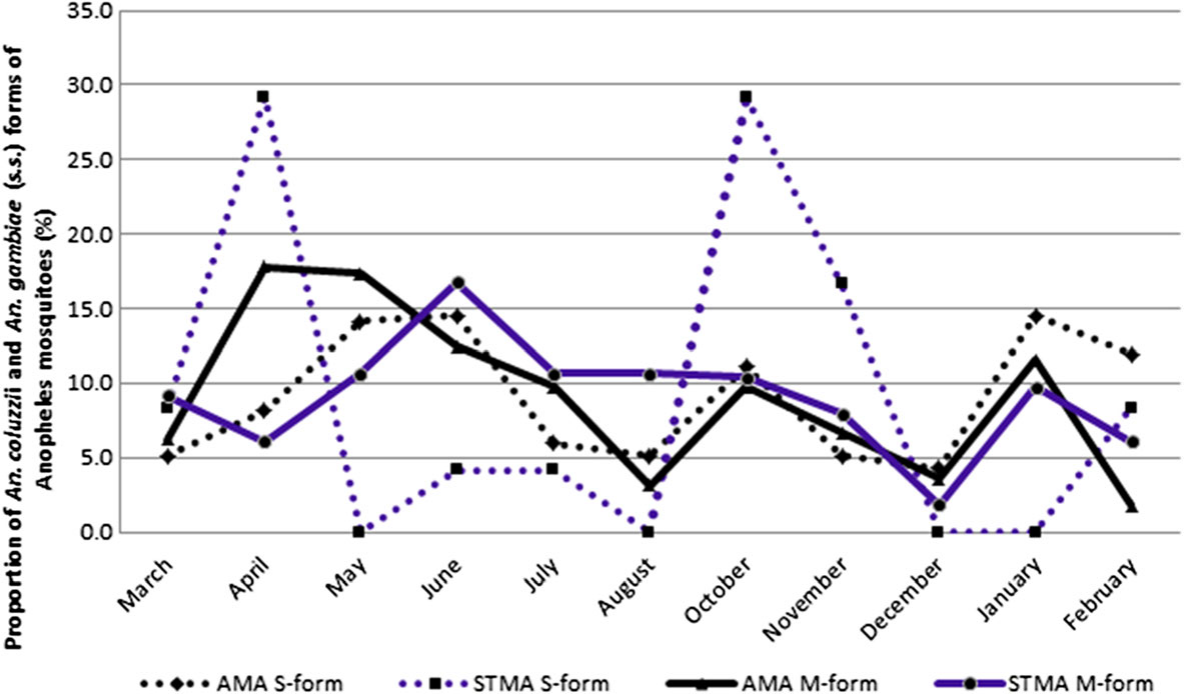
Monthly distribution of *An. coluzzii* and *An. gambiae* (*s.s.*) in Accra and Sekondi-Takoradi Metropolitan Areas

In Fig. 7, trends vary greatly for both *An. coluzzii* and *An. gambiae* (*s.s.*) when the two cities were examined separately. Generally, in AMA, *An. coluzzii* and *An. gambiae* (*s.s.*) varied throughout the sampling period. However, *An. coluzzii* were proportionally higher in March, April, May, July and November, compared to *An. gambiae* (*s.s.*) while the *An.gambiae* (*s.s.*) were proportionally higher in June, August, October, December, 2013, and January and February, 2014 compared to the *An. coluzzii*. In STMA, while the *An. gambiae* (*s.s.*) fluctuates greatly with high peaks in April and October, the *An. coluzzii* predominated in March, May, June, July, August, December, 2013 and January, 2014. *An. gambiae* (*s.s.*) were absent in May, August, December, 2013 and January, 2014. Comparing *An. coluzzii* of AMA and STMA showed that *An. coluzzii* were more abundant in AMA only in the months of April, May and December, 2013, while it was more abundant in months of June, July, August, October, November, 2013 and February, 2014 in STMA. Whenever *An. gambiae* (*s.s.*) of AMA increased, *An. gambiae* (*s.s.*) of STMA decreased. The STMA’s *An. gambiae* (*s.s.*) also has wide variations with the highest peaks in April and October.

Seasonal variations were also observed in the population of total *Anopheles* larvae sampled. As expected, the wet season had significantly higher proportion of *Anopheles* larvae compared to the dry season (*Z* = 8.3683, *P* < 0.0001), accounting for over 60% of all *Anopheles* mosquito larvae sampled from the two cities. Likewise, significantly higher proportions of larvae were observed in the wet season compared to the dry season in AMA (*Z* = 3.5859, *P* = 0.0003) and STMA (*Z* = 3.0641, *P* = 0.0022) (Table 3). Similar to the larval population, mean larval densities were significantly higher in the wet season compared to the dry season for combined study areas (t_(161)_ = 2.367, *P* = 0.0191) and AMA t_(75)_ = 2.324, *P* = 0.0228) (Table 3). However, in STMA, though mean larval density was higher in the wet season compared to the dry season, it was not significant t_(108)_ = 1.141, *P* = 0.2562).

**Table 3:** Proportion and density of larvae compared between wet and dry seasons in Accra and Sekondi-Takoradi Metropolitan Areas

	Season	AMA	STMA	ALL
% of total *Anopheles* larvae (number of habitats sampled)	Wet	69.2 (26)	62.2 (30)	66.4 (56)
	Dry	30.8 (20)	37.8 (18)	15.2 (38)
Mean larval density (per dip)	Wet	9.2 ± 1.7	3.1 ± 0.6	6.1 ± 0.9
	Dry	4.2 ± 1.1	2.1 ± 0.6	3.1 ± 0.6

*Abbreviations*: *AMA* Accra Metropolitan Area, *STMA* Sekondi-Takoradi Metropolitan Area

### Spatial distribution of *Anopheles* species

**Fig. 8:**
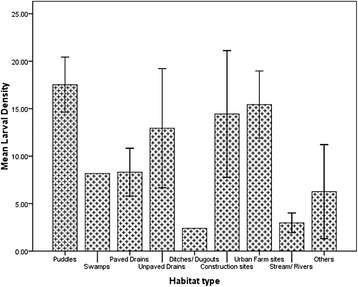
Habitat and larval density in the two study areas combined. The bars represent the mean larval density for habitat types found in the two cities. Error bars are the standard errors of the means

**Fig. 9:**
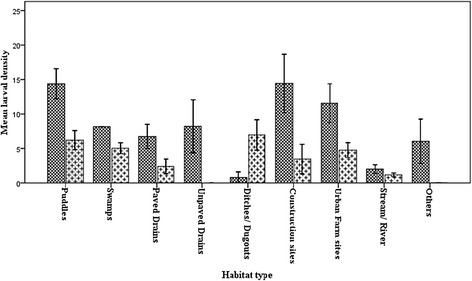
Habitat and larval density compared between Accra (AMA) and Sekondi- Takoradi Metropolitan Areas (STMA). The dark bars with square designs represent mean larval densities for Accra Metropolitan Area, the light bars with ‘plus’ signs indicate the mean larval density for Sekondi- Takoradi Metropolitan Area. Error bars are the standard errors of the means

Analysis of variance showed that the mean larval densities varied significantly among habitat types (ANOVA: *F*_(8,89)_ = 3.384, *P* = 0.0020) with puddles (13.7/dip) having the highest and stream/river the lowest (2.3/dip) (Fig. 8). Separate focus on each city showed similar significant variations in mean larval densities among habitat types in AMA (ANOVA: *F*_(8, 44)_ = 2.987, *P* = 0.0063) (Fig. 9). Though significant variations existed among the habitat types in STMA (ANOVA: *F*_(6,39)_ =2.555, *P* = 0.0342), it was due to low larval density of streams/rivers (1.5/dip), which was significantly lower as compared to the rest of the habitat types (all *P* < 0.05). No significant differences were found among the rest of the habitat types in STMA. Comparing AMA to STMA, showed that larval densities of habitat types in AMA were generally higher than those of STMA. Puddles and urban farm sites in AMA had significantly higher larval densities compared to the same habitats in STMA (*t*_(19)_ = 2.224, *P* = 0.0385 and *t*_(13)_ = 2.378, *P* = 0.0109, respectively).

**Fig. 10:**
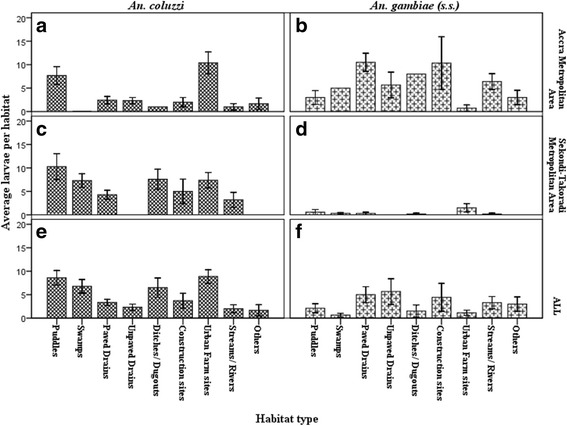
Habitat types and *An. coluzzii* and *An. gambiae s.s.* The dark bars with square designs in boxes (**a**, **c** and **e**) represent the average *An coluzzii* larvae in Accra and Sekondi-Takoradi Metropolitan Areas and a pooled from both cities respectively. The light bars with ‘plus’ signs in boxes (**b**, **d** and **f**) indicate the average *An gambiae* (*s.s.*) larvae in Accra and Sekondi-Takoradi Metropolitan Areas and a pooled from both cities. Error bars are the standard errors of the means

Significant variations were also observed in *An. coluzzii* and *An. gambiae* (*s.s.*) mean counts among the habitat types in both AMA and STMA (*P* < 0.05). Comparing the counts of *An. coluzzii* between AMA and STMA revealed that apart from puddles and urban farm sites, the rest of the habitat types in STMA had higher mean numbers compared to AMA (Fig. 10a,c). Conversely, *An. gambiae* (*s.s.*) mean counts were generally and interestingly higher in AMA compared to STMA (Fig. 10b,d). Additionally, puddles and urban farm sites that had higher mean numbers of *An. coluzzii* compared to the rest of the habitats had the lowest *An. gambiae* (*s.s.*) numbers in AMA (Fig. 10a,b). Four habitats including urban farms, puddles, swamps and ditches/ dugouts contributed approximately 70% of all *An. coluzzii* identified in the entire study whereas drains (paved and unpaved), construction sites, streams/rivers and “others” contributed 80% of all *An. gambiae* (*s.s.*) sampled in the two cities (Fig. 10e,f).

### Nature, origin and stability of habitats

*Anopheles* breeding sources were grouped as temporary and permanent. Overall, the analysis revealed that 62 (66%) of the 94 *Anopheles* habitats were permanent, when the two cities were combined. Similarly, sixty-five percent (65%) of all habitats found in AMA were permanent types (Table 4). These include puddles (19.6%), paved drains (15.2%), ditches/dugouts (2.2%), urban farms (13.0%) and streams/rivers (15.2%). All paved drains, ditches/ dugouts and streams/ rivers, were permanent habitats in AMA. However, unpaved drains, construction sites, swamps and “others” were all temporary in AMA. These habitats also constituted 2.2, 6.5, 6.5 and 6.5% respectively of all the temporary habitats in AMA. Most of the puddles (69%) and urban farm sites (75%) were permanent habitats in AMA.

**Table 4:** Nature and origin of *Anopheles* mosquito habitats in Accra Metropolitan Area

Habitat type	Nature of site		Origin of site	
	Temporary	Permanent	Natural	Man-made
	*n* (%)	*n* (%)	*n* (%)	*n* (%)
Puddles	4 (30.8)	9 (69.2)	0 (0)	14 (100)
	8.7	19.6	0	30.4
Swamps	1 (100)	0 (0)	0 (0)	0 (0)
	2.2	0	0	0
Paved drains	0 (0)	7 (100)	0 (0)	7 (100)
	0	15.2	0	15.2
Unpaved drains	3 (100)	0 (0)	0 (0)	3 (100)
	6.5	0	0	6.5
Ditches/ Dugouts	0 (0)	1 (100)	0 (0)	1 (100)
	0	2.2	0	2.2
Construction sites	3 (100)	0 (0)	0 (0)	3 (100)
	6.5	0	0	6.5
Urban farm sites	2 (25.0)	6 (75.0)	2 (25.0)	6 (75.0)
	4.3	13.0	4.3	13.0
Stream/ River beds	0 (0)	7 (100)	7 (100)	0 (0)
	0	15.2	15.2	0
Other	3 (100)	0 (0)	0 (0)	3 (100)
	6.5	0	0	6.5
No. of habitats (*n* = 46)	16	30	9	37
% of total	34.8	65.2	19.6	80.4

In STMA, 66.7% of the habitats sampled were permanent (Table 5). These were swamps (25.0%), paved drains (8.3%), ditches/dugouts (4.2%), construction sites (8.3%), urban farm sites (10.4%) and streams/rivers (10.4%). All construction sites and stream/ rivers were permanent habitats in STMA. Puddles found in STMA were all temporary and constituted about 15% of total habitats for STMA. Ditches/ dugouts in STMA were split equally 50% temporary and 50% permanent whereas paved drains were 43% temporary and 57% permanent.

**Table 5:** Nature and origin of *Anopheles* mosquito habitats in Sekondi-Takoradi Metropolitan Area

Habitat type	Nature of site		Origin of site	
	Temporary	Permanent	Natural	Man-made
	*n* (%)	*n* (%)	*n* (%)	*n* (%)
Puddles	7 (100)	0 (0)	0 (0)	7 (100)
	14.6	0	0	14.6
Swamps	1 (7.7)	12 (92.3)	10 (76.9)	3 (23.1)
	2.1	25.0	20.8	6.2
Paved drains	3 (42.9)	4 (57.1)	0 (0)	7 (100)
	6.2	8.3	0	14.6
Ditches/ Dugouts	2 (50.0)	2 (50.0)	0 (0)	4 (100)
	4.2	4.2	0	8.3
Construction sites	0 (0)	4 (100)	0 (0)	4 (100)
	0	8.3	0	8.3
Urban farm sites	3 (37.5)	5 (62.5)	0 (0)	8 (100)
	6.2	10.4	0	16.7
Stream/ River beds	0 (0)	5 (100)	5 (100)	0 (0)
	0	10.4	10.4	0
No. of habitats (*n* = 48)	16	32	15	33
% of total	33.3	66.7	31.2	68.8

The origin of *Anopheles* habitats was grouped into either natural or man-made. Overall, 70 (74.5%) of the 94 *Anopheles* mosquito habitats found were man-made and the remaining 25.5% were natural. About 80% of the habitats found in AMA were man-made and the rest natural. All stream/river habitats were naturally occurring *Anopheles* breeding places in AMA. In STMA, 69% of *Anopheles* habitats were man-made. Indeed, habitats such as puddles, paved drains, ditches/dugouts, construction sites and urban farm sites all owed their origin to human activities.

In terms of proportions, *Anopheles* mosquitoes produced by permanent breeding sites were 61.3% and that of temporary sites was 38.7% for all the two cities combined. In AMA, permanent breeding sites yielded 57.4% and the temporary sites had 42.6% of all *Anopheles* sampled in the city. In the case of STMA, permanent sites had 65% while temporary sites gave 35% of *Anopheles* mosquitoes sampled.

When the origin and nature of habitats were combined, further analysis revealed that 39 (55.7%) of the 70 man-made *Anopheles* habitats were permanent while 31 (44.3%) were temporary sites. Also 23 (95.8%) of the 24 natural *Anopheles* habitats were permanent with the remaining 1 (4.2%) being temporary habitats overall. In AMA, 21 (56.8%) of the 37 man-made habitats were permanent while 16 (43.2%) were temporary and all 9 (100%) of the natural habitats were permanent. Likewise, in STMA, 18 (54.5%) of 33 man-made habitats were permanent while 15 (45.5%) were temporary and 14 (93.3%) of 15 natural habitats were permanent with remaining 1 (6.7%) being temporary. In both cities, majority of the habitats were permanent and man-made.

## Discussion

Although about 83% of habitats identified were breeding places for different types of mosquitoes, *Anopheles* mosquito larvae were found in only 49% of them and the frequency of seeing a habitat harbouring *Anopheles* larvae in AMA was 1.7 times higher than in STMA. These variations may be due to the differences in the quality of water, that is, the physical, chemical and biological compositions of the water in the various habitats [25–27].

The data also revealed that *Anopheles* mosquito habitats were very diverse in the two cities. The diversity of the habitats is obviously due to the improper execution of developmental projects, lack of maintenance and poor environmental management that characterize many cities in developing countries. This is not the first time habitat types such as puddles, swamps, ditches/ dugouts, construction sites, urban farm sites, streams/ river edges and ponds/lagoons had been identified, other researchers had found them in urban areas [8, 28]. The two most common *Anopheles* mosquito habitat types found in this study were puddles (19%) and paved drains (19%) and this corroborates the earlier findings of De Silva & Marshall [29].

Puddles and urban farm sites in the two cities accounted for more than 51% of all *Anopheles* mosquitoes sampled and in AMA, the two habitats contributed a little over 60% of all *Anopheles* mosquitoes sampled. In STMA, however, swamps and urban farm sites were the highest contributors to *Anopheles* mosquitoes sampled in the city, accounting for approximately 53%. This indicates that eradicating puddles, and destroying breeding sites around urban farm sites and swamps can significantly reduce the population of *Anopheles* mosquitoes in the two cities.

Mosquito habitats have been classified as permanent and temporary. Grouping the habitats into temporary and permanent sites helps in understanding the stability of the breeding sites and for that matter, the extent to which each site contributes to the population of *Anopheles* mosquitoes in the study areas [30]. Temporary sites were mainly rain-dependent and dried up when it had not rained for a while. Permanent sites on the other hand, had a regular source of water either from underground as was in the case of certain low and marshy areas, rivers/streams or broken pipes which normally support puddles, as found also by Imbahale et al. [30] and Mereta et al. [31]. These mean whereas permanent habitats continuously support the breeding of *Anopheles* mosquitoes, breeding in temporary habitats is curtailed when they dry up due to intermittent supply of water. About 65 and 67% of habitats found in AMA and STMA, respectively, were permanent and as a result, 66% of all the habitats identified in the two cities put together were permanent. This explains why malaria transmission in cities is perennial [32]. *Anopheles* mosquito habitats were also classified by their origin. The data revealed that 74.5% of the habitats were man-made and the rest natural. Also, over 80 and 69% of the habitats in AMA and STMA, respectively, were man-made. These indicate that intense human activities in the urban milieu are creating breeding places for the *Anopheles* mosquitoes and are, therefore, major factors in malaria transmission. Interestingly, the data showed that more than half of the man-made habitats were permanent (55.7%) and the trend is the same for both cities. Though this is lower than what was observed in Kenya, where man-made habitats constituted about 95% of the breeding sources encountered [29, 33], it is still high. One reason why paved drains, puddles, ditches/dugouts were permanent breeding sites was that nobody attempted to clean the areas, repair broken pipes, remove debris from choked drains or fill the dugouts at construction sites that created those habitats. For example, a broken pipe that resulted in a puddle had been left unrepaired throughout the sampling period at one of the sampling point in AMA. Also a paved drain that had been clogged with debris in another sampling point in AMA was also left unattended to throughout the sampling period. In both AMA and STMA, numerous ditches/ dugouts produced by individuals, private companies and government agencies had all been left unattended to throughout the sampling period. These data support the suggestion that urban malaria is largely man-made [34] and can be reduced just by everybody doing the right thing.

Contrary to the findings of Khaemba et al. [33], Keating et al. [28] and Mereta et al. [31] showing that proportions of *Anopheles* mosquitoes produced by permanent sites were less than those of temporary sites, in this study higher proportion (61.3%) of *Anopheles* mosquitoes were found in permanent sites of the two study areas. The proportion is even higher (65%) in permanent sites of STMA than that of AMA (57.4%). This corroborates the findings of De Silva & Marshall [29] showing that *Anopheles* mosquitoes were most likely to be found in permanent shallow sunlit pools than temporary sites because the latter may not provide sufficient time for the maturation of *Anopheles* mosquitoes from eggs to adults. The implication is that malaria transmission would no longer be seasonal as observed by De Castro et al. [35] and Dery et al. [36] but perennial. It is also worth mentioning that on certain occasions and depending on the volume of water, permanent sites may either get too polluted or get flushed by heavy downpour and hence be rid of *Anopheles* larvae [37].

Over 99% of *Anopheles* mosquitoes sampled were morphologically identified as *An. gambiae* complex for the two cities combined, just as was found by [8] in AMA. Other *Anopheles* species such as *An. coustani* and *An. rufipes* were very rare and found only in STMA. It is known that *An. gambiae* complex is the dominant *Anopheles* species in Ghana [38] and this study has confirmed that. Similarly, almost all the members of the *An. gambiae* complex analysed were *An. coluzzii* and *An. gambiae* (*s.s.*) (together constituting over 99%) with only 0.2% being *An. melas*. Again, the few *An. melas* observed were found in STMA only. Additionally, members of the *An. gambiae* complex in AMA were made up of similar proportions of *An. coluzzii* and *An. gambiae* (*s.s.*)*.* These are not different from what others had found. *An. coluzzii* and *An. gambiae* (*s.s.*) have been reported as the most dominant species in Ghana and were the only members of the *Anopheles gambiae* complex in AMA [8, 39, 40]. The predominance of *An. coluzzii and An. gambiae* (*s.s.*) in Ghana is not surprising because they have been reported as the most widespread of all the malaria-transmitting mosquitoes in sub-Saharan Africa [41, 42].

The overall data showed predominance of *An. coluzzii* (62%) over the *An. gambiae* (*s.s.*) (29%) and STMA had an overwhelming prevalence of the *An. coluzzii* (86%), which is significantly higher than that of *An. gambiae* (*s.s.*). This agrees with the findings that suggest that *An. coluzzii* is more prevalent in savannah areas as compared to *An. gambiae* (*s.s.*) which is more dominant in forest zones [39, 43–45]. However, AMA had almost equal proportion of the *An. coluzzii* (44%) and *An. gambiae* (*s.s.*) (46%). Higher proportion of *An. gambiae* (*s.s.*) was found in similar studies in AMA [8, 46]. On the contrary, Kabula et al. [40] found more *An. coluzzii* than *An. gambiae* (*s.s.*) in AMA. There are, therefore, some variations in the reports of the proportions of *An. coluzzii* and *An. gambiae* (*s.s.*) in AMA and in the entire southern Ghana. This was also observed by Clarkson et al. [47] in mapping the frequencies of *An. coluzzii* and *An. gambiae* (*s.s.*) in southern Ghana. These variations can be attributed to the particular areas where sampling was done. For example, samples taken from Korle-Bu for this study, that of Achondu et al. [47] and Klinkernberg et al. [8] all had higher proportions of *An. coluzzii* than *An. gambiae* (*s.s.*) and all samples collected from Airport area by these same authors had more *An. gambiae* (*s.s.*) than *An. coluzzii*. However, samples taken by Kabula et al. [40] from Korle-Bu, Legon and Madina yielded 56.46% of *An. coluzzii* and 43.54% of *An. gambiae* (*s.s.*)*.*

Temporal distribution of *Anopheles* mosquitoes showed that both larval counts and density were high in June and October with dips in August and December. The data suggest that larval counts and density of *Anopheles* mosquitoes not only increase but also their breeding places increase tremendously during rainy months of the year. This is also in agreement with literature. Larval counts and density of *Anopheles* mosquitoes are known to be high during rainy seasons and decline during dry seasons [48–50]. This is obviously due to loss of some habitats and decline in mosquito populations during the dry season.

The overall results showed that *An. coluzzii* declined from March 2013 to February 2014 while the *An. gambiae* (*s.s.*) slightly increased within the same period. In AMA, both *An. coluzzii* and *An. gambiae* (*s.s.*) were observed throughout the year and their proportions were higher in the months of April, May, June, October and January. *Anopheles gambiae* (*s.s.*) was, however, absent from STMA in the months of May, August, December and January but with higher proportions in the months of April and October. This is similar to what Villarreal-Trevino et al. [51] found in Chiapas, Mexico where *An. darlingi* mosquito was more prevalent in certain months, for example, from June to September and with peaks in July but were less in other months.

Seasonal variations were also observed in the population of *Anopheles* larvae and larval densities in the study areas, with wet season having both higher larval population and mean larval density compared to the dry season. This suggests that any action to reduce malaria transmission may have to be intensified during the rainy season [52, 53].

Analysis of variance showed that the mean larval densities varied significantly among habitat types with puddles having the highest. Ndenga et al. [54] found puddles to be the most productive larval habitats in Kenya. The high larval density together with the frequency of occurrence accounted for the high contribution of puddles to the total larvae sampled. Similar variations in larval densities were observed in AMA. Apart from streams/rivers which had lowest larval density compared to the rest of the habitat types, no significant differences were found among the rest of the habitat types in STMA. This indicates that whereas some habitats may be more important than others in AMA, almost all habitats in STMA are important in mosquito control. However, it is not clear what accounted for the high productivity of habitats in AMA in terms of larval density compared to STMA. For puddles and urban farm sites in AMA had significantly higher larval densities compared to the same habitats in STMA. The general low larval densities of habitats in STMA might be due to the quality of the water which is being discussed in a subsequent paper. But Kibret et al. [55] also found that some habitats were more important in serving as breeding grounds for *Anopheles* mosquitoes than others in Ethiopia.

Mean counts of *An. coluzzii* and *An. gambiae* (*s.s.*) also varied significantly among the habitat types in both cities. The similar high counts of *An. coluzzii* in puddles and urban farm sites in both cities might be due to similar physico-chemical factors prevailing in the habitats. This is also being looked at in our subsequent paper. Similar reasons may account for the high mean count of *An. gambiae* (*s.s.*) in AMA compared to STMA. Additionally, puddles and urban farm sites that had higher mean numbers of *An. coluzzii* compared to the rest of the habitats had the lowest *An. gambiae* (*s.s.*) numbers in AMA. In Mali, Edilo et al. [56] observed differences in habitat preference between the two species of *An. coluzzii* and *An. gambiae* (*s.s.*)*.* They found that while *An. gambiae* (*s.s.*) together with *An. arabiensis* exploit the same breeding habitats, *An. coluzzii* prefer different habitat from the two, though *An. coluzzii* and *An. gambiae* (*s.s.*) were also sympatric.

Regarding spatial distribution, mean larval densities in the two cities showed that puddles (13.7/dip), unpaved drains (12.9/dip) and urban farm sites (11.6/dip) had that highest larval density. However, construction sites, ditches/dugouts and urban farms were the three habitats with high larval densities in STMA whereas puddles, urban farms and construction sites constitute the highest larval habitats in AMA. It is interesting to know that the major contributors to mosquito population in both cities were man-made habitats, supporting the findings of Gimning et al. [57] and Imbahale et al. [30]; and hence suggesting that if we change the way things are done *Anopheles* population can be drastically reduced.

In AMA puddles and urban farm sites accounted for 63% of all *An. coluzzii* found in the city. This preference of *An. coluzzii* for puddles and urban farm sites is not clear and needs to be investigated. *Anopheles gambiae* (*s.s.*) was almost evenly distributed among the habitats in AMA, showing no preference for any habitat. However, 80% of *An. coluzzii* found in STMA were almost evenly distributed among nearly all habitats including puddles and urban farm sites which were their preferred habitats in AMA. *Anopheles gambiae* (*s.s.*) was generally low in proportion compared to *An. coluzzii* in all the habitats in STMA, however, urban farm sites alone contributed about half (49%) of all *An. gambiae* (*s.s.*) found in the city. This is not only interesting for *An. gambiae* (*s.s.*) control but also for *An. gambiae* (*s.s.*) larvae collection in STMA. Overall, 70% of all *An. coluzzii* sampled in the two cities were found in urban farms, puddles, swamps and ditches/ dugouts whereas, 80% of all *An. gambiae* (*s.s.*) sampled in the two cities were found in drains (paved and unpaved), construction sites, streams/ rivers and “others”. In contrast, Edilo et al. [56] did not find any association between the frequencies of larvae of *An. coluzzii* and *An. gambiae* (*s.s.*) among larval breeding habitats.

## Conclusions

In summary, this study reveals the types of *Anopheles* mosquitoes, their spatio-temporal distribution and preferred habitats in urban areas of southern Ghana. Both larval density and counts were high in wet seasons and more associated with puddles, urban farms and drains. These have prospects for targeted interventions to manage, reduce or eliminate *Anopheles* breeding habitats. The study also suggests the need for city-dwellers to change their attitude with regard to environmental management. This is because majority of the permanent habitats were man-made and over 60% of all the habitats encountered were permanent. Proper environmental management is therefore key to curtailing the perennial breeding of *Anopheles* mosquitoes and for that matter malaria transmission in the two cities.

## Additional file

Additional file 1: **a** Species identification. Lane 1: ladder; Lane 2: positive; Lanes 3 to 11 and 13 to 21: *Anopheles gambiae* at 390 bp; Lane 12: *Anopheles melas* at 464 bp. **b** PCR-RFLP-molecular identification. Lane 1: ladder; Lanes 2, 3, 5, 9, 11: *Anopheles gambiae *(*s.s*.); Lanes 4, 6, 7, 8, 10, 12: *Anopheles coluzzii*. (TIF 165 kb)

## Abbreviations

AMA: Accra metropolitan area
ANOVA: Analysis of variance
EC: Electrical conductivity
SPSS: Statistical package for social sciences
SSA: sub-Saharan Africa
STMA: Sekondi-takoradi metropolitan area
