# Supplementary figures and images for: Diversity in breeding sites and distribution of Anopheles mosquitoes in selected urban areas of southern Ghana
Source: Parasit Vectors. 2017 Jan 13;10:25. doi: 10.1186/s13071-016-1941-3 (PMC5237286; doi:10.1186/s13071-016-1941-3)

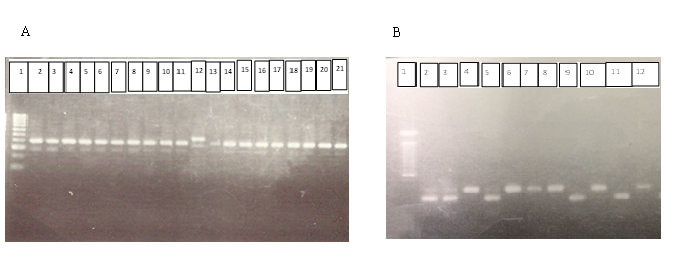

Supplement: Additional file 1: — a Species identification. Lane 1: ladder; Lane 2: positive; Lanes 3 to 11 and 13 to 21: Anopheles gambiae at 390 bp; Lane 12: Anopheles melas at 464 bp. b PCR-RFLP-molecular identification. Lane 1: ladder; Lanes 2, 3, 5, 9, 11: Anopheles gambiae (s.s.); Lanes 4, 6, 7, 8, 10, 12: Anopheles coluzzii. (TIF 165 kb) [file 13071_2016_1941_MOESM1_ESM.tif]
